# Spectral, Information-Theoretic and Thermodynamic Properties of a Fractal Position-Dependent Mass Schrödinger System

**DOI:** 10.3390/nano16130787

**Published:** 2026-06-23

**Authors:** Q. R. D. S. Moreira, L. F. Ximenes, A. R. P. Moreira, D. M. Neves, J. B. R. Silva, J. C. Nascimento

**Affiliations:** 1 Departamento de Engenharia de Teleinformática, Universidade Federal do Ceará, Campus do Pici, Bloco 725, Fortaleza 60455-760, CE, Brazil; 2 Secretaria da Educação do Estado do Ceará (SEDUC-CE), Coordenadoria Regional de Desenvolvimento da Educação (CREDE 6), Sobral 62039-010, CE, Brazil; 3 Secretaria da Educação do Estado do Ceará (SEDUC-CE), Coordenadoria Regional de Desenvolvimento da Educação (CREDE 9), Horizonte 62880-384, CE, Brazil; 4 Postgraduate Program in Electrical and Computer Engineering, Federal University of Ceará, Sobral 62010-560, CE, Brazil; 5 CTI Center for Information Technology Renato Archer, Campinas 13069-901, SP, Brazil

**Keywords:** position-dependent mass, fractal Schrödinger equation, information-theoretic measures, Shannon entropy, Fisher information, Fisher–Shannon complexity, thermodynamic properties

## Abstract

In this work, we investigate the spectral, information-theoretic, and thermodynamic properties of a fractal Schrödinger system with position-dependent mass subject to an effective semiconductor-like confinement. We employ a fractal momentum operator and a Von Roos Hamiltonian with BenDaniel–Duke ordering to obtain exact analytical solutions for the energy spectrum and wave functions. The interplay between the fractal parameter α, the effective lattice scale $l_{0}$, and the harmonic confinement strength ω is explored. We perform a comprehensive analysis of the Shannon entropy, Fisher information, and Fisher–Shannon complexity in both coordinate and momentum spaces. Our results demonstrate that these parameters directly control the localization–delocalization transition and the informational architecture of the quantum states, while satisfying the entropic and Fisher uncertainty relations. Furthermore, we derive the exact partition function and the corresponding thermodynamic properties (free energy, internal energy, entropy, and specific heat) of the system. The analytical framework presented offers valuable insights into the spectral, information-theoretic, and thermodynamic behavior of quantum systems in fractal semiconductor-like environments.

## 1. Introduction

The study of quantum systems with position-dependent mass (PDM) has attracted considerable attention due to its wide applicability in describing the physics of semiconductor heterostructures [1], quantum dots [2], nanocrystals [3], and other low-dimensional systems with spatial inhomogeneities [4,5]. In these effectively confined structures, the charge carriers experience a varying effective mass that strongly modifies the spectral and localization properties of the system. Concurrently, the exploration of fractal geometries in quantum mechanics has provided a powerful framework to model irregular, anisotropic, and complex media where standard Euclidean dynamics are insufficient [6]. The integration of PDM formalisms with fractional or fractal calculus opens new avenues for understanding the behavior of quantum particles in realistic, highly structured materials [7]. In this context, effective mass models are widely used as continuum descriptions of carrier dynamics in semiconductor nanostructures, where spatial confinement typically occurs at the nanometer scale. In such systems, the effective mass approximation remains valid provided that the variation of material parameters occurs smoothly over characteristic lengths comparable to quantum confinement scales in low-dimensional devices.

Position-dependent mass (PDM) models have become increasingly important in the theoretical description of nanomaterials, semiconductor heterostructures, quantum dots, and low-dimensional quantum systems, where spatial inhomogeneities strongly modify the effective dynamics of charge carriers [7,8,9,10,11,12,13]. In such systems, the effective mass may vary due to compositional gradients, lattice deformations, impurity distributions, or external interactions, making the PDM formalism a realistic framework for describing electronic transport, confinement effects, and spectral properties [14,15,16,17]. Recent investigations have shown that PDM approaches are also relevant in the study of oscillator-like quantum wells, anisotropic quantum wires, semiconductor nanostructures, and nonuniform heterostructures [18,19,20]. Furthermore, generalized PDM formulations have been successfully applied to systems involving fractal geometries, fractional quantum mechanics, noncommutative effects, and complex mass distributions, opening new possibilities for modeling realistic nanoscale environments with irregular spatial structures [6,21,22,23,24,25,26,27,28,29,30]. These developments demonstrate that combining PDM quantum mechanics with fractal or non-Euclidean frameworks provides a powerful theoretical tool for investigating localization phenomena, quantum confinement, and informational properties in advanced semiconductor and nanostructured materials.

A deeper understanding of such complex quantum systems can be achieved through information-theoretic measures, which provide complementary information beyond the energy spectrum and wave-function structure [31]. Information-theoretic measures, such as Shannon entropy [32], Fisher information [33], and Fisher–Shannon complexity [34], have proven to be exceptionally sensitive tools for characterizing the spatial spreading, local gradients, and internal organization of quantum states [35,36,37,38,39]. While Shannon entropy provides a global measure of delocalization and uncertainty [40], Fisher information acts as a local descriptor sensitive to the rapid oscillations of the probability density [41]. Together, they offer a complete informational footprint of the quantum system in both coordinate and momentum spaces, complementing the traditional Heisenberg uncertainty principle [42].

In this paper, we propose and exactly solve a position-dependent mass fractal Schrödinger equation featuring an effective semiconductor-like confinement. We consider a power-law mass profile and a fractal harmonic-like potential that model the characteristics of a crystal lattice with damping. After determining the exact energy spectrum and the corresponding wave eigenfunctions, we perform a detailed analysis of the Shannon entropy, Fisher information, and Fisher–Shannon complexity. We systematically investigate how the fractal dimension, the lattice scale, and the confinement strength govern the information distribution and the localization–delocalization transitions. Finally, exploiting the equally spaced nature of the obtained energy spectrum, we evaluate the canonical partition function and derive closed-form expressions for the thermodynamic state variables of the system.

In the present work, the expression “semiconductor-like” refers to an effective quantum framework that reproduces some fundamental characteristics commonly found in semiconductor nanostructures. In particular, the model incorporates a position-dependent effective mass, a characteristic lattice-scale parameter, and a confining interaction leading to discrete bound states. These ingredients are frequently employed in the theoretical description of quantum wells, quantum dots, heterostructures, and graded semiconductor materials. Therefore, the proposed system should be interpreted as a semiconductor-inspired effective model rather than a microscopic band-structure description of a specific material. It is important to emphasize that the present model is not intended as a microscopic band-structure description of a particular semiconductor material. Rather, it should be interpreted as a semiconductor-inspired effective framework in which the position-dependent mass, the characteristic length scale $l_{0}$, and the fractal deformation of the kinetic operator are employed as phenomenological ingredients to investigate the combined effects of confinement, mass inhomogeneity, and non-Euclidean geometry on the spectral, information-theoretic, and thermodynamic properties of quantum states. Consequently, $l_{0}$ should be understood as an effective characteristic length of the medium rather than a literal atomic lattice constant.

The paper is organized as follows. In Section 2, we introduce the position-dependent mass fractal Schrödinger equation, define the effective mass and potential profiles, and obtain the exact analytical solutions. In Section 3, we present the definitions of the information-theoretic measures and discuss the numerical results for the Shannon entropy, Fisher information, and Fisher–Shannon complexity in conjugate spaces. Section 4 is devoted to the derivation of the thermodynamic properties. Finally, our concluding remarks are presented in Section 5.

## 2. Position-Dependent Mass Fractal Schrodinger Equation

For a fractal dimension corresponding to 0<α≤1 in the *x* direction, we can describe the motion of a particle confined to an infinitely thin, frictionless string, which is tightly stretched between two impenetrable walls separated by a distance *l*, using the fractal momentum operator and the Hamiltonian:

(1) $$\begin{array}{cc} {\hat{p}}^{\left(\alpha \right)} & =-\;i\hbar {\left(\frac{l_{0}}{l-x}\right)}^{\alpha -1}\nabla , \end{array}$$

(2) $$\begin{array}{cc} {\hat{H}}^{\left(\alpha \right)} & =-\frac{\hbar^{2}}{2m_{e}\left(x\right)}\;\left[{\left(\frac{l_{0}}{l-x}\right)}^{2(\alpha -1)}\;\;\Delta +\frac{\alpha -1}{l_{0}}{\left(\frac{l_{0}}{l-x}\right)}^{2\alpha -1}\;\;\nabla \right]+\frac{i\hbar }{2}\frac{m_{e}^{\prime }\left(x\right)}{m_{e}^{2}\left(x\right)}{\hat{p}}^{\left(\alpha \right)}\\ \; & \;+V\left(x\right)+U_{\eta ,\xi ,\zeta }^{\mathrm{PDM}}\left(x\right), \end{array}$$

where $U_{\eta ,\xi ,\zeta }^{\mathrm{PDM}}\left(x\right)$ is the Von Roos potential emerging from PDM, given by Roos [4]:

$$U_{\eta ,\xi ,\zeta }^{\mathrm{PDM}}\left(x\right)=\frac{\xi +1}{4}\;\frac{m_{e}^{\prime \prime }\left(x\right)}{m_{e}^{2}\left(x\right)}+\frac{\eta \zeta -\xi -1}{2m_{e}^{3}\left(x\right)}\;m_{e}^{\prime }{\left(x\right)}^{2}.$$

For the BenDaniel–Duke ordering, the parameters in the Von Roos Hamiltonian are chosen as [5]

η=0,ξ=−1,ζ=0,

which satisfy the constraint η+ξ+ζ=−1.

Substituting these values into the Von Roos potential, one obtains

$$U_{\eta ,\xi ,\zeta }^{\mathrm{PDM}}\left(x\right)=0.$$

Thus, the Schrödinger fractal equation in 1D becomes

(3) $$\psi^{\prime \prime }\left(x\right)+\left[\frac{\alpha -1}{l-x}-{\left(\frac{l_{0}}{l-x}\right)}^{1-\alpha }\frac{m_{e}^{\prime }}{m_{e}}\right]\psi^{\prime }\left(x\right)+{\left(\frac{l_{0}}{l-x}\right)}^{2(1-\alpha )}\frac{2m_{e}}{\hbar^{2}}\left(E-V\right)\;\psi \left(x\right)=0.$$

Now we can define the mass profile of the model. Power-law profiles are used to describe quantum dots and nanocrystals, as well as being used in studies of diatomic molecules and the phenomenon of vibrational resonance [7,43,44]. We chose a profile in power-law form:

(4) $$m_{e}\left(x\right)=m{\left(\frac{l-x}{l_{0}}\right)}^{2(\alpha -1)},$$

where *m* has mass dimensions and describes the intensity of the effective mass, and $l_{0}$ represents the equilibrium spacing of neighboring atoms. We make this choice because the effective mass profile and the characteristic length scale $l_{0}$ mimic some of the essential features of semiconductor nanostructures. In this context, $l_{0}$ plays the role of an effective lattice parameter associated with the underlying medium, while the position-dependent mass models the spatial variation of carrier dynamics commonly observed in heterostructures and graded semiconductor systems. For this reason, we refer to the proposed framework as a semiconductor-like system, emphasizing that it represents an effective description rather than a microscopic model of a specific semiconductor material.

The effective-mass profile adopted in this work is not intended to reproduce a specific experimental heterostructure. Instead, it provides a flexible parameterization that allows us to investigate how spatial inhomogeneity, fractal effects, and confinement mechanisms influence the quantum and informational properties of the system. Such effective descriptions are commonly used in the theoretical study of semiconductor nanostructures where the carrier mass varies due to changes in composition, strain, or interface effects.

**Figure 1:**
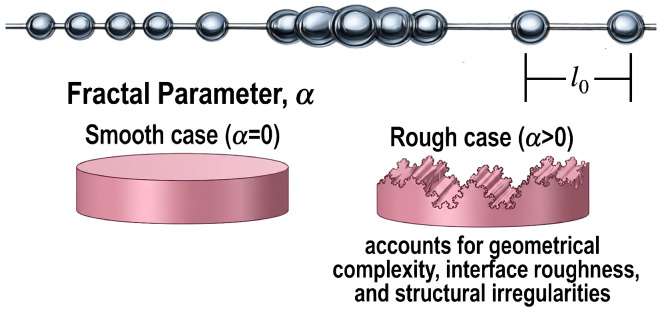
Schematic representation of the physical interpretation of the proposed model. The position-dependent effective mass $m_{e}\left(x\right)$ mimics carrier-mass variations commonly encountered in graded semiconductor heterostructures. The characteristic length scale $l_{0}$ represents an effective nanoscale confinement length, while the fractal parameter α accounts phenomenologically for geometrical complexity, interface roughness, and structural irregularities.

From a nanoscience perspective, the proposed effective-mass profile may be viewed as a simplified continuum representation of carrier dynamics in semiconductor nanosystems exhibiting spatially varying composition or confinement. Examples include compositionally graded quantum wells, strained heterostructures, core–shell nanowires, and semiconductor quantum dots, where the effective mass changes due to variations in band structure across the device [45,46,47,48,49,50]. In this interpretation, the parameter $l_{0}$ defines a characteristic nanoscale length associated with confinement or interface regions, whereas the fractal parameter α introduces an effective description of structural complexity and nonuniformity (see Figure 1). Although the present model is not intended to reproduce a specific material system, it captures generic mechanisms governing localization, confinement, and information transport in low-dimensional semiconductor nanostructures.

**Figure 2:**
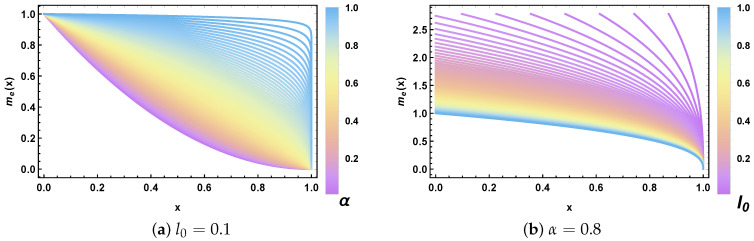
Effective mass profile for m=l=1.

Figure 2 illustrates the behavior of the effective mass profile $m_{e}\left(x\right)$ for fixed l=1 under variations of the fractal parameter α and the lattice scale parameter $l_{0}$. The analytical expression (4) is directly responsible for the trends observed in the numerical plots, ensuring full consistency between the formula and Figure 2. In Figure 2a, for fixed $l_{0}=0.1$, the effective mass decreases monotonically as the coordinate *x* approaches the boundary x→l for 0<α<1, as expected from the negative exponent 2(α−1)<0. The decay rate is strongly controlled by the fractal parameter α, with smaller values of α producing a stronger divergence of the factor ${\left(l-x\right)}^{2(\alpha -1)}$ and thus a more pronounced suppression of the effective mass. As α→1, the exponent approaches zero and the mass profile becomes spatially uniform, recovering the Euclidean limit. In Figure 2b, for fixed α=0.8, the parameter $l_{0}$ acts as a global scaling factor of the effective mass profile. Larger values of $l_{0}$ reduce the overall amplitude of $m_{e}\left(x\right)$ through the prefactor $l_{0}^{-2(\alpha -1)}$, while preserving the same spatial dependence, leading to a uniform modification of the mass distribution over the whole domain. This behavior is fully consistent with the analytical expression and confirms that $l_{0}$ controls the characteristic scale of the effective medium associated with the semiconductor-like structure. Physically, these results demonstrate that the interplay between the fractal parameter and the lattice scale generates a tunable position-dependent mass profile, in agreement with the analytical formulation, which can significantly influence the localization properties, spectral structure, and informational behavior of the quantum system.

Applying the transformation $\psi \left(x\right)=e^{{\left(\frac{l-x}{l_{0}}\right)}^{\alpha }}\phi \left(x\right)$ reduces (3) to the form:

(5) $$\phi^{\prime \prime }+\frac{\alpha -1}{l-x}\;\phi^{\prime }+\left[\frac{2m}{\hbar^{2}}\left(E-V\right)-\frac{\alpha -1}{l_{0}^{2}}{\left(\frac{l-x}{l_{0}}\right)}^{\alpha -3}-\frac{{\left(\alpha -1\right)}^{2}}{l_{0}^{2}}{\left(\frac{l-x}{l_{0}}\right)}^{2\alpha -4}\right]\phi =0.$$

We need to choose the shape of the potential. Since our purpose is to study the behavior of a semiconductor crystal lattice, we choose a fractal potential that describes a quantum mechanical system with damping [7]:

(6) $$V\left(x\right)=\frac{\hbar^{2}}{2m}\left[\omega \;{\left(l-x\right)}^{2}-\frac{\alpha -1}{l_{0}^{2}}{\left(\frac{l-x}{l_{0}}\right)}^{\alpha -3}-\frac{{\left(\alpha -1\right)}^{2}}{l_{0}^{2}}{\left(\frac{l-x}{l_{0}}\right)}^{2\alpha -4}\right].$$

To ensure dimensional consistency of the model, we briefly clarify the physical dimensions and roles of the main parameters. The quantity $l_{0}$ represents a characteristic length scale of the system and therefore has dimensions of length. The parameter ω plays the role of an effective confinement strength associated with the harmonic-like potential. In order for the potential term

(7) $$V\left(x\right)∼\frac{\hbar^{2}}{2m}\;\omega {\left(l-x\right)}^{2}$$

to be dimensionally consistent with energy, ω must carry dimensions of inverse length squared (or, equivalently, energy divided by $\hbar^{2}$ times mass), ensuring that all terms in the Hamiltonian have dimensions of energy. With these identifications, one verifies that the Schrödinger equation, the energy spectrum, and all thermodynamic quantities are dimensionally consistent throughout the analysis.

Substituting into (5) obtained

(8) $$\Delta^{\left(\alpha \right)}\phi +\frac{\alpha -1}{l-x}\;\nabla^{\left(\alpha \right)}\phi +\left(\frac{2m}{\hbar^{2}}E-\omega {\left(\frac{l-x}{l_{0}}\right)}^{2}\right)\phi =0.$$

For completeness of the derivation and to address possible concerns regarding intermediate steps, we provide in Appendix A a detailed calculation showing how Equation (3) is systematically transformed into Equation (7). In particular, Appendix A explicitly presents the action of the fractal momentum operator, the origin of the additional derivative terms, and the role of the BenDaniel–Duke ordering in eliminating the von Roos contribution. This detailed derivation ensures full transparency of the formal manipulations and clarifies how the final differential equation is obtained in a consistent and self-contained manner.

We can apply the ansatz

(9) $$\phi \left(x\right)={\left(l-x\right)}^{\frac{\alpha }{2}-1}\;e^{-\frac{\sqrt{\omega }}{2}\frac{{\left(l-x\right)}^{2}}{l_{0}}}\;w\;\left(z\right),\;z:=\sqrt{\omega }\frac{{\left(l-x\right)}^{2}}{l_{0}},$$

transforming Equation (5) into a confluent hypergeometric equation, whose regular solution is a combination of U(a,b,z) and $L_{n}^{\left(\nu \right)}\left(z\right)$. The boundary condition ϕ(l)=0 fixes the quantization, leading to the linear spectrum

(10) $$E_{n}=\frac{2\sqrt{\omega }\;\hbar^{2}}{m\;l_{0}^{2}\;\alpha }\left(n+\frac{2-\alpha }{2}\right),\;n=1,2,3,\ldots$$

and to the wave eigenfunctions

(11) $$\phi_{n}\left(x\right)\propto {\left(l-x\right)}^{\frac{\alpha }{2}-1}\exp\;\left[-\frac{\sqrt{\omega }}{2}\frac{{\left(l-x\right)}^{2}}{l_{0}}\right]L_{n}^{\left(\alpha /2\right)}\;\left(\sqrt{\omega }\frac{{\left(l-x\right)}^{2}}{l_{0}}\right).$$

The eigenfunction contains the prefactor ${\left(l-x\right)}^{\frac{\alpha }{2}-1}$, which appears to diverge as x→l for 0<α<1. However, the full wave function remains physically acceptable due to the interplay between this algebraic factor and the exponential damping term. Indeed, near the boundary x→l, one has

(12) $$|\phi_{n}{\left(x\right)|}^{2}∼{\left(l-x\right)}^{\alpha -2}\exp\;\left[-\frac{\sqrt{\omega }}{l_{0}}{\left(l-x\right)}^{2}\right].$$

Introducing the change of variable $y={\left(l-x\right)}^{2}$, the normalization integral becomes

(13) $$\int_{0}^{l}{\left|\phi_{n}\left(x\right)\right|}^{2}dx∼\int_{0}^{\epsilon }y^{\frac{\alpha }{2}-1}e^{-cy}dy,$$

with $c=\sqrt{\omega }/l_{0}>0$. This integral is convergent for all 0<α≤1, ensuring that the eigenfunctions are square-integrable and therefore normalizable.

Furthermore, the boundary condition ϕ(l)=0 is satisfied in the limiting sense since

(14) $$\lim_{x\to l}{\left(l-x\right)}^{\frac{\alpha }{2}-1}e^{-\frac{\sqrt{\omega }}{2}\frac{{\left(l-x\right)}^{2}}{l_{0}}}=0,$$

in the distributional sense of square-integrable functions, which is sufficient for the self-adjointness of the Hamiltonian in the considered Hilbert space.

The energy spectrum given by Equation (10) exhibits a linear dependence on the quantum number *n*, which is characteristic of harmonic-type confined systems. However, in the present model, the spacing between adjacent energy levels is strongly modified by the combined effects of the fractal parameter α, the lattice scale $l_{0}$, and the confinement strength ω. In particular, the factor $\sqrt{\omega }$ increases the overall energy scale, showing that stronger external confinement leads to more energetic bound states. On the other hand, the inverse quadratic dependence on $l_{0}$ indicates that larger equilibrium spacing between neighboring atoms reduces the energy spectrum, which can be interpreted as a weakening of the effective confinement generated by the semiconductor-like lattice structure. The fractal parameter α plays a particularly important role, since the spectrum is inversely proportional to α, implying that stronger fractal effects (smaller α) significantly enhance the energy levels and increase the separation between neighboring states. Furthermore, the term (2−α)/2 introduces a fractal correction to the ground-state energy, demonstrating that the non-Euclidean geometry directly modifies the vacuum structure of the system. Physically, these results reveal that the interplay between fractality, position-dependent mass distribution, and harmonic confinement provides an effective mechanism for controlling the spectral properties of semiconductor-like quantum systems.

## 3. Information-Theoretic Measures

In this section, we investigate the information-theoretic properties of the system through the analysis of Shannon entropy, Fisher information, and Fisher–Shannon complexity in both coordinate and momentum representations. These quantities provide complementary insights into the localization, spreading, and internal structure of quantum states in conjugate spaces. For simplicity, we set l=ℏ=1.

For a normalized quantum state described by the wave function $\psi_{n}\left(x\right)$, the probability density in configuration space is defined by [35,36]

(15) $$\rho_{n}\left(x\right)={\left|\psi_{n}\left(x\right)\right|}^{2},$$

which characterizes the probability of locating the particle around the position *x*. The corresponding Shannon entropy in position space is expressed as [51]

(16) $$S_{x}^{\left(n\right)}=-\int \rho_{n}\left(x\right)\;\ln\;\left[\rho_{n}\left(x\right)\right]dx,$$

and quantifies the degree of delocalization of the state in coordinate space.

The momentum-space wave function is obtained from the Fourier transform of $\psi_{n}\left(x\right)$, leading to the probability density

(17) $$\gamma_{n}\left(p\right)={\left|\psi_{n}\left(p\right)\right|}^{2}.$$

A relevant point concerns the use of the standard Fourier transform to define the momentum-space wave function. In the present formulation, the fractal effects and the position-dependent mass modify the configuration-space dynamics through the Hamiltonian operator and the effective differential structure, but they do not alter the underlying canonical commutation relations between position and momentum. As a consequence, the momentum representation is obtained through the usual unitary Fourier transform, which maps the position-space wave function into its conjugate representation without requiring a modified kernel or deformed measure. The Hilbert space structure remains standard, and the fractal corrections are fully encoded in the form of the eigenfunctions in coordinate space. Therefore, the use of the ordinary Fourier transform is consistent within the effective quantum-mechanical framework adopted in this work and provides a well-defined momentum-space probability density.

Accordingly, the Shannon entropy in momentum space is given by

(18) $$S_{p}^{\left(n\right)}=-\int \gamma_{n}\left(p\right)\;\ln\;\left[\gamma_{n}\left(p\right)\right]dp.$$

These two entropic measures are linked through the entropic uncertainty relation introduced by Beckner, Bialynicki-Birula, and Mycielski (BBM), namely [52,53]

(19) $$S_{x}^{\left(n\right)}+S_{p}^{\left(n\right)}\geq D\left(1+\ln\pi \right),$$

which establishes a nonzero lower bound for the total information entropy. In the present work, the system is effectively one-dimensional, such that D=1. It is important to emphasize that the application of the BBM entropic uncertainty relation in the present framework remains valid despite the presence of a finite spatial domain, a position-dependent mass, and a fractal deformation of the coordinate system. The validity follows from the fact that the probability densities in both position and momentum representations are properly normalized within the corresponding Hilbert space. In particular, the position-space wave functions are square-integrable under the modified measure induced by the fractal coordinate transformation, ensuring a well-defined Shannon entropy. On the other hand, the momentum-space wave functions are constructed through a unitary Fourier-type transform acting on the physical Hilbert space, which preserves normalization and guarantees the conjugate structure between position and momentum variables. Therefore, although the underlying geometry modifies the functional form of the wave functions, it does not affect the fundamental assumptions required for the BBM inequality. Consequently, the entropic uncertainty relation remains applicable without modification, and all entropy calculations reported in this work are mathematically consistent and physically well-defined.

To obtain a more detailed description of the spatial distribution of information, one may also consider the local entropy densities in position and momentum spaces, respectively:

(20) $$\begin{array}{ccc} \rho_{s}^{\left(n\right)}\left(x\right) & = & -\rho_{n}\left(x\right)\;\ln\;\left[\rho_{n}\left(x\right)\right],\\ \rho_{s}^{\left(n\right)}\left(p\right) & = & -\gamma_{n}\left(p\right)\;\ln\;\left[\gamma_{n}\left(p\right)\right]. \end{array}$$

These functions measure the local contribution of each region of the probability distribution to the total Shannon entropy, allowing a more refined characterization of localization effects and information concentration.

In analogy with Shannon entropy, Fisher information is also fundamentally associated with the probability distribution of a physical system. Within quantum mechanics, this connection emerges naturally from the probability densities constructed from the wave functions in coordinate and momentum spaces. The corresponding Fisher information densities are defined by [37,54]

(21) $$\begin{array}{ccc} \rho_{I}^{\left(n\right)}\left(x\right) & = & \rho_{n}\left(x\right){\left[\frac{d}{dx}\ln\left[\rho_{n}\left(x\right)\right]\right]}^{2},\\ \rho_{I}^{\left(n\right)}\left(p\right) & = & \gamma_{n}\left(p\right){\left[\frac{d}{dp}\ln\left[\gamma_{n}\left(p\right)\right]\right]}^{2}. \end{array}$$

For continuous probability distributions, the Fisher information measures in position and momentum spaces are, respectively, given by [37,54]

(22) $$\begin{array}{ccc} I_{x}^{\left(n\right)} & = & \int \rho_{n}\left(x\right){\left[\frac{d}{dx}\ln\left[\rho_{n}\left(x\right)\right]\right]}^{2}dx,\\ I_{p}^{\left(n\right)} & = & \int \gamma_{n}\left(p\right){\left[\frac{d}{dp}\ln\left[\gamma_{n}\left(p\right)\right]\right]}^{2}dp, \end{array}$$

where both quantities satisfy the positivity conditions $I_{x}^{\left(n\right)}>0$ and $I_{p}^{\left(n\right)}>0$.

Furthermore, the uncertainty relation for Fisher information is [55]

(23) $$I_{x}I_{p}\geq 4.$$

These expressions establish a direct connection between quantum uncertainty and the informational content encoded in the probability densities [38,39].

Finally, we analyze the Fisher–Shannon complexity, which combines global and local information measures to quantify the structural complexity of the quantum system. This quantity is defined as [56]

(24) $$\begin{array}{c} C_{\mathrm{IS}}^{\left(x,p\right)}=J_{\left(x,p\right)}\;I_{\left(x,p\right)}, \end{array}$$

where

(25) $$\begin{array}{c} J_{\left(x,p\right)}=\frac{1}{2\pi e}\;\exp\;\left(\frac{2}{D}S_{\left(x,p\right)}\right), \end{array}$$

and $J_{\left(x,p\right)}$ denotes the Shannon entropy power. For D=1, the Fisher–Shannon complexity satisfies the inequality

(26) $$C_{\mathrm{IS}}^{\left(x,p\right)}\geq 1.$$

These quantities provide a useful framework for understanding the interplay between localization, oscillatory behavior, and informational complexity in quantum systems.

The numerical results presented in Table 1, Table 2 and Table 3 reveal that the fractal parameter α, the characteristic lattice scale $l_{0}$, and the confinement strength ω play a crucial role in determining the informational behavior of the position-dependent mass fractal system. From Table 1, one observes that increasing α leads to an increase in the position-space Shannon entropy $S_{x}$, while the momentum entropy $S_{p}$ decreases. This behavior indicates that the wave packet becomes progressively more delocalized in coordinate space and more localized in momentum space as the fractal dimension approaches the standard Euclidean regime. Simultaneously, the total entropy $S_{x}+S_{p}$ decreases slightly, but always remains above the BBM lower bound, confirming the consistency of the entropic uncertainty relation. In Table 2, the increase in the lattice parameter $l_{0}$ enhances $S_{x}$ and suppresses $S_{p}$, showing that larger equilibrium atomic spacing induces stronger spatial spreading of the quantum states, which can be interpreted as a reduction in effective confinement in semiconductor-like fractal lattices. Moreover, higher excited states exhibit larger total entropy, reflecting the increasing oscillatory structure and spatial extension of the eigenfunctions. Table 3 shows that increasing the oscillator parameter ω produces the opposite effect: the position entropy becomes smaller, even negative for strongly localized states, whereas $S_{p}$ increases. Physically, this demonstrates that stronger harmonic confinement compresses the probability density in coordinate space and transfers uncertainty to momentum space, in full agreement with the complementarity principle. Altogether, these results show that the interplay between fractality, effective-mass distribution, and external confinement directly controls the localization–delocalization transition of the quantum states, providing a useful informational characterization of fractal semiconductor systems with position-dependent mass.

**Figure 3:**
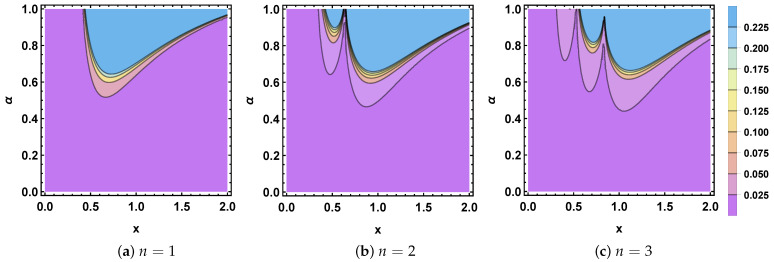
Entropy density for $m=l_{0}=\omega =0.1$.

Figure 3 illustrates the behavior of the Shannon entropy density in coordinate space for different quantum states n=1,2,3 with fixed parameters $l_{0}=\omega =0.1$. One observes that the entropy density exhibits a highly nonuniform spatial distribution, strongly influenced by both the fractal parameter α and the excitation level of the system. For small values of α, the entropy density remains weakly distributed over the spatial domain, indicating a higher degree of localization of the probability density. As α increases, the entropy density becomes more pronounced and extends over a wider region, revealing the progressive delocalization of the quantum states induced by the fractal deformation of the system. In addition, higher excited states display more intricate spatial structures and sharper variations in the entropy density, reflecting the increasing oscillatory nature of the wave functions and the larger informational content associated with excited modes. The appearance of localized peaks and abrupt transitions near specific spatial regions suggests that the position-dependent mass profile and the fractal geometry generate preferential localization zones, where the information content becomes strongly concentrated.

**Figure 4:**
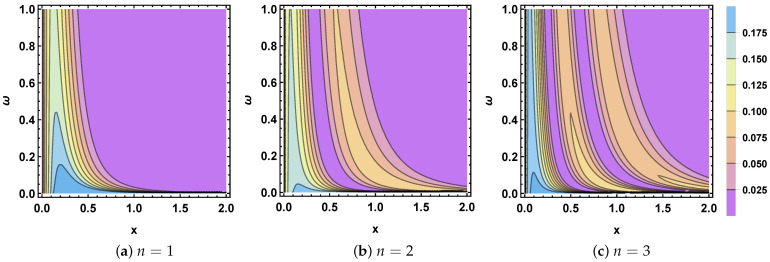
Entropy density for m=α=ω=0.1.

In Figure 4, corresponding to the case α=ω=0.1, the entropy density shows a strong dependence on the effective lattice scale $l_{0}$. For small values of $l_{0}$, the entropy density becomes highly concentrated near the origin, indicating strong localization of the quantum states due to the reduced equilibrium spacing between neighboring atoms. As $l_{0}$ increases, the entropy distribution spreads over a wider spatial region, revealing the gradual delocalization of the probability density induced by the weakening of the effective confinement associated with the position-dependent mass profile. This effect becomes more pronounced for higher excited states, whose entropy landscapes exhibit increasingly complex structures and additional localized regions, reflecting the richer oscillatory behavior of the corresponding wave functions.

**Figure 5:**
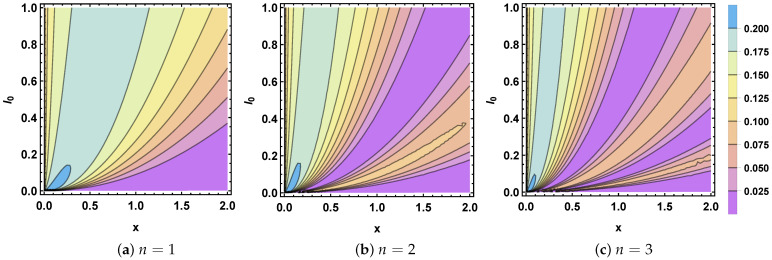
Entropy density for $m=\alpha =l_{0}=0.1$.

Figure 5, obtained for $\alpha =l_{0}=0.1$, illustrates the influence of the oscillator parameter ω on the entropy density. One observes that increasing ω produces a stronger concentration of the entropy density around small spatial regions, which is consistent with the enhancement of the harmonic confinement. In particular, the contour lines become progressively compressed as ω grows, indicating that the wave functions become more localized in coordinate space. Moreover, higher excited states display multiple localized structures and sharper entropy gradients, signaling the emergence of increasingly intricate informational patterns. Physically, these results demonstrate that both the effective lattice geometry and the external confining interaction play a fundamental role in controlling the spatial distribution of quantum information in fractal position-dependent mass systems. The combined effects of fractality, confinement, and effective mass deformation therefore provide a mechanism for tuning localization properties and informational organization in semiconductor-like quantum structures.

The numerical results displayed in Table 4, Table 5 and Table 6 provide important information about the localization properties and uncertainty structure of the fractal position-dependent mass system through the Fisher information measures. From Table 4, it is observed that increasing the fractal parameter α systematically decreases the position-space Fisher information $I_{x}$, while the momentum-space Fisher information $I_{p}$ increases significantly. Since Fisher information is highly sensitive to local variations of the probability density, this behavior indicates that larger values of α produce smoother and more delocalized wave functions in coordinate space, whereas the corresponding momentum distributions become more sharply localized. In addition, the product $I_{x}I_{p}$ grows with both α and the quantum number *n*, showing that highly excited states possess stronger oscillatory structures and greater informational content in conjugate spaces. All values satisfy the inequality $I_{x}I_{p}\geq 4$, confirming the validity of the Fisher-based uncertainty relation.

Table 5 shows that increasing the lattice scale parameter $l_{0}$ strongly suppresses $I_{x}$ and enhances $I_{p}$. Physically, this behavior reflects the fact that larger equilibrium spacing between neighboring atoms weakens the effective confinement generated by the position-dependent mass profile, leading to broader spatial distributions and consequently lower local sensitivity in coordinate space. At the same time, the momentum distribution becomes more concentrated, which explains the rapid growth of $I_{p}$. The increase in the product $I_{x}I_{p}$ for larger $l_{0}$ indicates that the deformation of the effective lattice geometry enhances the overall informational complexity of the quantum states.

Finally, Table 6 reveals that the oscillator strength ω produces the opposite effect. As ω increases, the position-space Fisher information rises substantially, while $I_{p}$ decreases. This behavior indicates that stronger harmonic confinement generates highly localized probability densities in coordinate space, increasing the gradient content of the wave functions and consequently enhancing the Fisher information. At the same time, the momentum-space distribution becomes smoother and less localized. The large values of the Fisher-information product $I_{x}I_{p}$ observed for strong confinement regimes therefore reflect a more pronounced localization structure and a richer uncertainty distribution between the conjugate representations, rather than quantum correlations. In this sense, the Fisher-information product provides a sensitive measure of the informational complexity and local fluctuations of the quantum state. Altogether, these results demonstrate that the interplay between fractality, effective mass distribution, and external confinement governs the local informational structure of the system, allowing precise control of localization properties and uncertainty characteristics in fractal semiconductor-like environments.

**Figure 6:**
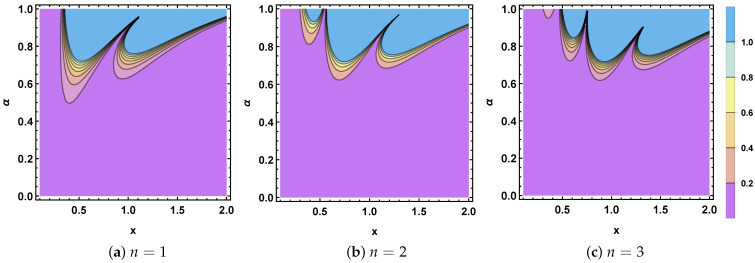
Information density for $m=l_{0}=\omega =0.1$.

Figure 6 presents the Fisher information density in coordinate space for different quantum states n=1,2,3 with fixed parameters $l_{0}=\omega =0.1$. The contour profiles reveal that the informational distribution is strongly affected by both the fractal parameter α and the excitation level of the system. For small values of α, the Fisher information density remains relatively weak and localized in restricted spatial regions, indicating smoother probability distributions with reduced local gradients. As α increases, the information density develops pronounced peaks and sharper contour structures, particularly near specific spatial regions where the probability density varies rapidly. Since Fisher information is highly sensitive to local fluctuations of the wave function, these intense peaks indicate regions of strong localization and enhanced spatial sensitivity of the quantum states. Furthermore, the increase in the quantum number *n* introduces additional localized structures and more intricate contour patterns, reflecting the growing oscillatory character of the excited-state wave functions. The emergence of multiple high-information regions demonstrates that excited states possess richer local informational content and stronger spatial correlations.

**Figure 7:**
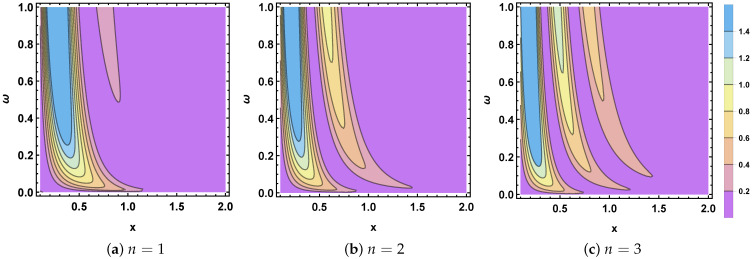
Information density for m=α=ω=0.1.

**Figure 8:**
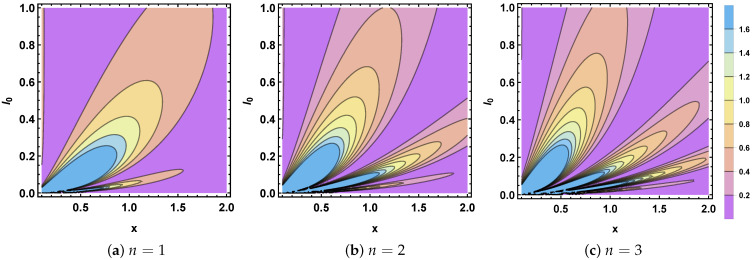
Information density for $m=\alpha =l_{0}=0.1$.

In Figure 7, corresponding to the case α=ω=0.1, the information density exhibits highly localized structures concentrated near small spatial regions for low values of $l_{0}$. This behavior indicates strong localization of the probability density and large spatial gradients of the wave functions, which are characteristic features of high Fisher information. As $l_{0}$ increases, the localized peaks broaden and shift toward larger spatial regions, revealing that the increase in the effective lattice spacing weakens the confinement generated by the position-dependent mass profile. Moreover, higher excited states develop additional localized structures and more intricate contour patterns, reflecting the enhanced oscillatory character and the richer local informational content of the corresponding wave functions. Figure 8, obtained for $\alpha =l_{0}=0.1$, shows that the oscillator parameter ω strongly modifies the spatial organization of the Fisher information density. For increasing ω, the contour lines become progressively compressed and highly concentrated around narrow spatial regions, demonstrating the enhancement of localization induced by the stronger harmonic confinement. The emergence of elongated high-information regions and multiple localized peaks for larger quantum numbers reveals that excited states possess increasingly complex local structures and stronger sensitivity to spatial variations. Physically, these results indicate that the combined effects of the fractal geometry, the effective-mass distribution, and the confining interaction govern the local information content of the system. Consequently, the model provides a mechanism for controlling quantum localization and informational sensitivity in fractal semiconductor-like environments through the tuning of the lattice scale and external confinement parameters.

The results presented in Table 7, Table 8 and Table 9 describe the behavior of the Fisher–Shannon complexity, which combines the global spreading measured by the Shannon entropy with the local sensitivity encoded in the Fisher information. Consequently, this quantity provides a refined characterization of the internal organization and structural complexity of the quantum states in both coordinate and momentum spaces. In all cases, the values satisfy the inequality $C_{\mathrm{IS}}^{\left(x,p\right)}\geq 1$, confirming the consistency of the informational framework adopted for the fractal position-dependent mass system.

From Table 7, one observes that the position-space complexity $C_{\mathrm{IS}}^{x}$ generally increases with both the fractal parameter α and the quantum number *n*. This behavior indicates that higher fractal dimensions and more excited states produce probability densities with richer internal structure, characterized by stronger oscillatory behavior and a more intricate balance between localization and delocalization. In momentum space, however, the complexity exhibits a nontrivial behavior, reflecting the competition between the reduction in Shannon entropy and the enhancement of Fisher information as α increases. This demonstrates that the fractal deformation strongly modifies the informational organization of the conjugate momentum states.

Table 8 shows that increasing the lattice scale parameter $l_{0}$ enhances the Fisher–Shannon complexity in coordinate space, particularly for higher excited states. Physically, this means that larger equilibrium atomic spacing generates broader and more structured wave functions, increasing the internal complexity of the spatial probability distribution. In momentum space, the complexity reaches very large values, especially for excited states, indicating the emergence of highly nonuniform momentum distributions with pronounced informational correlations. These results reveal that the effective geometry induced by the position-dependent mass profile significantly affects the informational architecture of the system.

Finally, Table 9 demonstrates that increasing the oscillator strength ω produces a gradual growth of the position-space complexity while generally reducing the momentum-space complexity. This behavior reflects the stronger confinement induced by the harmonic interaction, which increases the local organization and gradient structure of the spatial wave functions while simultaneously smoothing the momentum distributions. The persistence of relatively large values of $C_{\mathrm{IS}}^{p}$ even for strong confinement indicates that the momentum-space states preserve a high degree of informational richness. Altogether, these results show that the combined effects of fractality, effective-mass distribution, and external confinement govern the structural complexity of the quantum states, providing deeper insight into the informational properties of fractal semiconductor-like quantum systems.

## 4. Thermodynamic Properties

In this section, we investigate the thermodynamic properties of the fractal position-dependent mass quantum system described by the discrete energy spectrum

(27) $$E_{n}=\frac{2\sqrt{\omega }\hbar^{2}}{ml_{0}^{2}\alpha }\left(n+\frac{2-\alpha }{2}\right),\;n=1,2,3,\ldots .$$

The linear dependence of the spectrum on the quantum number allows an exact analytical treatment of the canonical thermodynamic quantities. Since the parameters α, $l_{0}$, and ω directly modify the level spacing, they also govern the thermal behavior of the system.

Within the canonical ensemble formalism, the partition function is defined by

(28) $$Z=\sum_{n=1}^{\infty }e^{-\beta E_{n}},\;\beta =\frac{1}{k_{B}T},$$

where $k_{B}$ denotes the Boltzmann constant and *T* is the absolute temperature. Substituting the spectrum given in Equation (27), the partition function can be evaluated exactly, yielding

(29) $$Z\left(T\right)=\frac{\exp\left[-\frac{\left(3-\alpha \right)\sqrt{\omega }\hbar^{2}}{ml_{0}^{2}\alpha k_{B}T}\right]}{2\sinh\left[\frac{\sqrt{\omega }\hbar^{2}}{ml_{0}^{2}\alpha k_{B}T}\right]}.$$

To ensure full consistency between the analytical formulation and the numerical results, we emphasize that all thermodynamic quantities presented in this work are directly derived from the closed-form partition function Z(β) constructed from the exact energy spectrum. In particular, the entropy, internal energy, and specific heat are not obtained through independent numerical fitting procedures, but rather computed using the standard thermodynamic relations applied to Z(β). This guarantees that the plotted behaviors are fully constrained by the analytical structure of the model. Moreover, we have explicitly verified that the high-temperature regime (β→0) reproduces the expected asymptotic expansion of the partition function, leading to a logarithmic of the entropy, a linear classical-like growth of the internal energy, and a saturation of the specific heat, in agreement with the density-of-states analysis. This confirms the internal consistency between the closed-form expressions and the thermodynamic plots presented in the manuscript.

The Helmholtz free energy is obtained from the standard thermodynamic relation

(30) $$F=-k_{B}T\ln Z,$$

which leads to

(31) $$F=\frac{\left(3-\alpha \right)\sqrt{\omega }\hbar^{2}}{ml_{0}^{2}\alpha }+k_{B}T\ln\left[2\sinh\left(\frac{\sqrt{\omega }\hbar^{2}}{ml_{0}^{2}\alpha k_{B}T}\right)\right].$$

This quantity measures the available thermodynamic work and encodes the combined effects of fractality, confinement strength, and effective lattice geometry on the thermal equilibrium of the system.

The mean internal energy is calculated through the canonical expression

(32) $$U=-\frac{\partial }{\partial \beta }\ln Z,$$

resulting in

(33) $$U=\frac{\left(3-\alpha \right)\sqrt{\omega }\hbar^{2}}{ml_{0}^{2}\alpha }+\frac{\sqrt{\omega }\hbar^{2}}{ml_{0}^{2}\alpha }\coth\left(\frac{\sqrt{\omega }\hbar^{2}}{ml_{0}^{2}\alpha k_{B}T}\right).$$

The first contribution corresponds to the effective ground-state energy shifted by the fractal correction, while the second term describes the thermal excitation of the quantum states.

The canonical entropy is obtained from

(34) $$S=k_{B}\left(\ln Z+\beta U\right),$$

which gives

(35) $$S=k_{B}\left[\frac{\sqrt{\omega }\hbar^{2}}{ml_{0}^{2}\alpha k_{B}T}\coth\left(\frac{\sqrt{\omega }\hbar^{2}}{ml_{0}^{2}\alpha k_{B}T}\right)-\ln\left[2\sinh\left(\frac{\sqrt{\omega }\hbar^{2}}{ml_{0}^{2}\alpha k_{B}T}\right)\right]\right].$$

This expression characterizes the degree of thermal disorder and the distribution of accessible quantum states as the temperature varies.

Finally, the specific heat at constant volume is defined by

(36) $$C_{V}={\left(\frac{\partial U}{\partial T}\right)}_{V},$$

leading to

(37) $$C_{V}=k_{B}{\left(\frac{\sqrt{\omega }\hbar^{2}}{ml_{0}^{2}\alpha k_{B}T}\right)}^{2}{\mathrm{csch}}^{2}\left(\frac{\sqrt{\omega }\hbar^{2}}{ml_{0}^{2}\alpha k_{B}T}\right).$$

The specific heat provides important information about the thermal response of the system and the role played by the fractal parameter, the effective lattice spacing, and the confinement strength in the storage and transfer of thermal energy.

**Figure 9:**
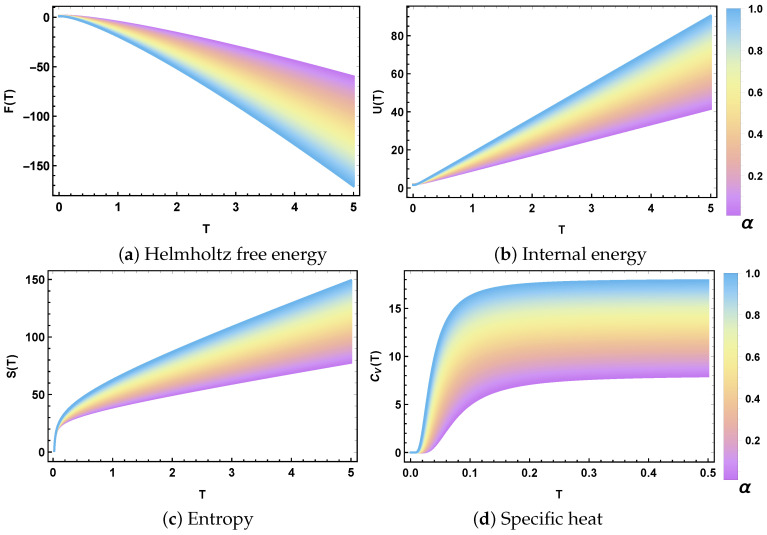
Thermodynamic properties with $m=l_{0}=\omega =0.1$.

Figure 9 illustrates the thermodynamic behavior of the fractal position-dependent mass system for fixed parameters $l_{0}=\omega =0.1$ and different values of the fractal parameter α. In Figure 9a, the Helmholtz free energy decreases monotonically with temperature, becoming more negative as *T* increases. This behavior indicates that thermal fluctuations progressively stabilize the system, while larger values of α intensify the decrease in the free energy due to the enhancement of the accessible thermal states induced by the fractal geometry. Figure 9b shows that the internal energy grows almost linearly with temperature, reflecting the continuous thermal excitation of the equally spaced quantum levels. Moreover, larger values of α lead to higher internal energy, revealing that the fractal deformation effectively increases the thermal capacity of the system. In Figure 9c, the entropy exhibits a monotonic increase with temperature, as expected from the increasing number of accessible microstates. The stronger growth of the entropy for larger α demonstrates that the fractal parameter enhances the informational disorder and the thermal spreading of the quantum states. Finally, Figure 9d presents the specific heat, which rapidly increases at low temperatures and subsequently approaches a saturation plateau. This behavior is characteristic of confined quantum systems with discrete spectra and indicates the existence of a finite number of thermally active states at low energies. The increase in the saturation value with α shows that the fractal geometry amplifies the thermal response of the system, allowing a larger storage of thermal energy.

**Figure 10:**
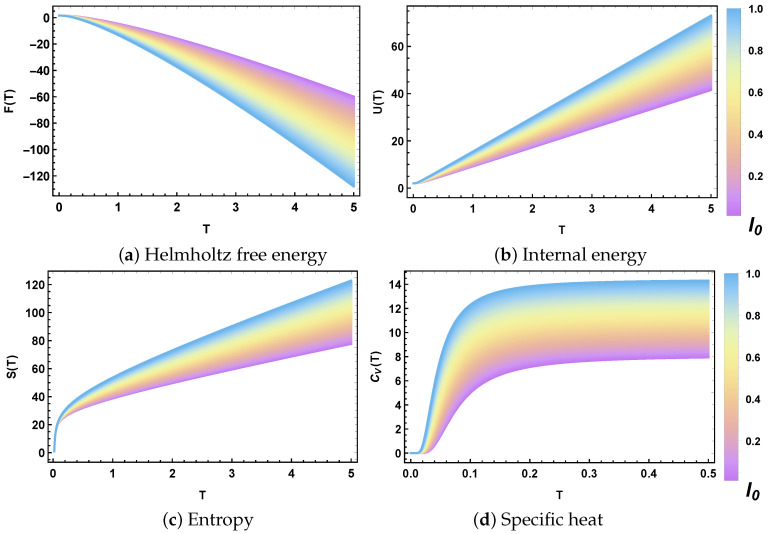
Thermodynamic properties with m=α=ω=0.1.

The same is observed by varying the parameter $l_{o}$ and fixing ω=α=0.1 (see Figure 10).

**Figure 11:**
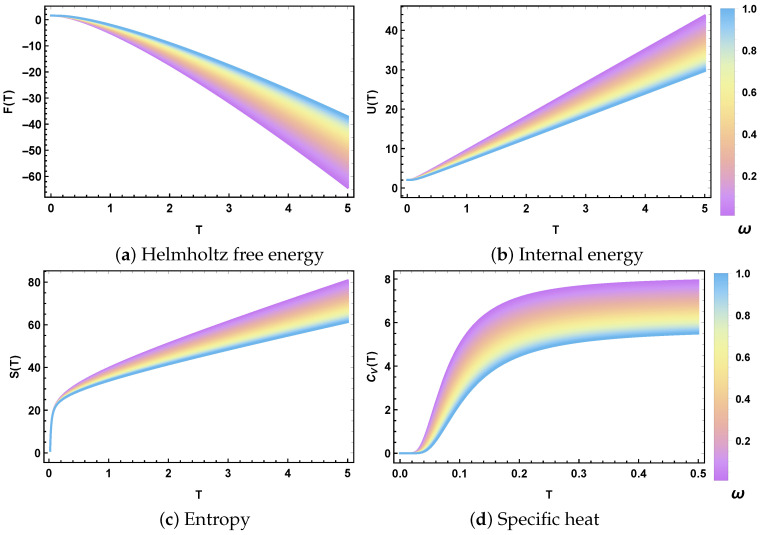
Thermodynamic properties with $m=l_{0}=\alpha =0.1$.

Figure 11 presents the thermodynamic properties of the fractal position-dependent mass system for fixed parameters $l_{0}=\alpha =0.1$ and different values of the oscillator strength ω. In Figure 11a, the Helmholtz free energy decreases monotonically with temperature, becoming more negative as the thermal energy increases. One observes that larger values of ω intensify the reduction in the free energy, indicating that stronger harmonic confinement enhances the thermodynamic stability of the system by increasing the spacing between the quantum energy levels. Figure 11b shows that the internal energy increases approximately linearly with temperature, reflecting the progressive thermal excitation of the discrete spectrum. However, increasing ω reduces the magnitude of the internal energy, since stronger confinement suppresses the thermal accessibility of higher excited states. In Figure 11c, the entropy grows monotonically with temperature, as expected from the increase in the number of accessible microstates. Nevertheless, larger values of ω lead to smaller entropy values, revealing that the stronger confining interaction restricts the spreading of the quantum states and reduces the thermal disorder of the system. Finally, Figure 11d illustrates the behavior of the specific heat, which rapidly increases in the low-temperature regime and subsequently approaches a saturation plateau. This behavior is characteristic of quantum systems with discrete spectra and finite thermal activation. The reduction in the saturation value with increasing ω demonstrates that stronger confinement suppresses thermal fluctuations and decreases the capacity of the system to store thermal energy.

## 5. Conclusions

In this work, we investigated a fractal Schrödinger equation with position-dependent mass in the framework of a semiconductor-like quantum system subjected to harmonic confinement. By introducing a fractal momentum operator and a power-law effective-mass profile, we obtained an exactly solvable model whose spectral and informational properties are strongly governed by the interplay between the fractal parameter α, the lattice scale parameter $l_{0}$, and the oscillator strength ω.

Using the BenDaniel–Duke ordering prescription, the effective potential associated with the position-dependent mass was eliminated, allowing the fractal Schrödinger equation to be reduced to a solvable differential equation. Through an appropriate transformation and ansatz, exact analytical expressions for the energy spectrum and wave functions were derived in terms of Laguerre polynomials. The resulting spectrum exhibits a linear dependence on the quantum number, characteristic of harmonic-type systems, but with level spacing strongly modified by the fractal geometry and the effective lattice structure. In particular, decreasing α enhances the energy levels and enlarges the separation between adjacent states, while increasing $l_{0}$ weakens the effective confinement and reduces the spectrum. The oscillator parameter ω controls the overall energy scale, producing stronger confinement for larger values.

We also analyzed the information-theoretic properties of the system through the Shannon entropy, Fisher information, and Fisher–Shannon complexity in both coordinate and momentum spaces. The numerical results demonstrated that increasing α or $l_{0}$ generally promotes delocalization in coordinate space, increasing the position-space Shannon entropy while reducing the corresponding Fisher information. Conversely, increasing the confinement strength ω compresses the wave functions, leading to stronger localization, smaller position entropy, and larger Fisher information. In all cases, the BBM entropic uncertainty relation and the Fisher-information uncertainty inequalities were fully satisfied, confirming the consistency of the quantum-informational description. The Fisher–Shannon complexity revealed that the combined effects of fractality, effective-mass distribution, and external confinement generate highly structured informational patterns, especially for excited states, where increasingly intricate oscillatory and localization effects emerge.

The thermodynamic properties of the model were investigated exactly within the canonical ensemble formalism. Closed analytical expressions were obtained for the partition function, Helmholtz free energy, internal energy, entropy, and specific heat. The results showed that the free energy decreases monotonically with temperature, whereas the internal energy and entropy increase due to the thermal activation of the discrete spectrum. The specific heat exhibits the characteristic saturation behavior of confined quantum systems with finite thermal accessibility. Furthermore, the fractal parameter α and the lattice scale $l_{0}$ enhance the thermal response of the system, increasing the entropy and the specific-heat saturation plateau, while larger values of ω suppress thermal fluctuations and reduce the storage of thermal energy.

Beyond the formal analytical results, the present model provides an effective theoretical framework for describing quantum nanosystems with spatially varying effective mass and engineered confinement profiles. In particular, it can be interpreted as a simplified description of semiconductor heterostructures, quantum wells, nanowires, and quantum dots, where spatial inhomogeneities, interface effects, and finite-size confinement play a central role in the electronic properties. The introduction of the fractal parameter α offers an additional degree of freedom to model deviations from ideal crystalline order, including structural irregularities and effective geometrical complexity at the nanoscale. Therefore, the present work establishes a direct conceptual bridge between fractal quantum mechanics and realistic semiconductor nanostructures, reinforcing its relevance for quantum nanosystems and low-dimensional materials.

From an application-oriented perspective, the present results may be relevant for the theoretical modeling of semiconductor nanosystems where effective-mass engineering and quantum confinement are central design mechanisms. Examples include quantum wells, quantum dots, nanowires, superlattices, and compositionally graded heterostructures. In such systems, the ability to tune localization properties through the effective-mass distribution, confinement strength, and geometrical complexity is directly related to carrier transport, optical response, and quantum-information processing. Consequently, the exact analytical framework developed here may provide useful guidance for future investigations of nanoscale semiconductor devices and engineered low-dimensional quantum materials.

## Figures and Tables

**Table 1:** Numerical Shannon entropy for $m=l_{0}=\omega =0.1$.

*n*	α	$S_{x}$	$S_{p}$	$S_{x}+S_{p}$
1	0.1	−0.07868	2.96465	2.88596
	0.3	0.07757	2.63536	2.71293
	0.5	0.20580	2.30332	2.50912
2	0.1	0.14542	3.10448	3.24990
	0.3	0.34521	2.82170	3.16691
	0.5	0.40580	2.70394	3.10974
3	0.1	0.27349	2.95547	3.22896
	0.3	0.49366	2.71636	3.21002
	0.5	0.89838	2.28919	3.18757

**Table 2:** Numerical Shannon entropy for m=α=ω=0.1.

*n*	$l_{0}$	$S_{x}$	$S_{p}$	$S_{x}+S_{p}$
1	0.1	−0.07868	2.96465	2.88596
	0.2	0.26582	2.71353	2.97935
	0.4	0.61036	2.57848	3.18885
2	0.1	0.14542	3.10448	3.24990
	0.2	0.48712	2.78195	3.26907
	0.4	0.98906	2.36133	3.35039
3	0.1	0.27349	2.95547	3.22896
	0.2	0.61341	2.85863	3.47204
	0.4	0.95346	2.59412	3.54758

**Table 3:** Numerical Shannon entropy for $m=\alpha =l_{0}=0.1$.

*n*	ω	$S_{x}$	$S_{p}$	$S_{x}+S_{p}$
1	0.1	−0.07868	2.96465	2.88596
	0.5	−0.48344	3.10282	2.61938
	1.0	−0.65775	3.22065	2.56290
2	0.1	0.14542	3.10448	3.24990
	0.5	−0.26259	3.12513	2.86254
	1.0	−0.43827	3.19469	2.75642
3	0.1	0.27349	2.95547	3.22896
	0.5	−0.13658	3.00866	2.87208
	1.0	−0.31313	3.14483	2.83170

**Table 4:** Numerical Fisher information for $m=l_{0}=\omega =0.1$.

*n*	α	$I_{x}$	$I_{p}$	$I_{x}I_{p}$
1	0.1	6.4262	5.8528	37.6117
	0.3	5.7340	6.6431	38.0917
	0.5	4.7704	9.8707	47.0876
2	0.1	6.8256	6.9266	47.2776
	0.3	5.8979	8.6050	50.7516
	0.5	3.6762	16.8692	62.0157
3	0.1	7.2003	9.3161	67.0799
	0.3	6.0557	12.2037	73.9028
	0.5	3.6448	25.9525	94.5938

**Table 5:** Numerical Fisher information for m=α=ω=0.1.

*n*	$l_{0}$	$I_{x}$	$I_{p}$	$I_{x}I_{p}$
1	0.1	6.4262	5.8528	37.6117
	0.2	3.2658	12.8529	41.9749
	0.4	1.7333	29.1410	50.5129
2	0.1	6.8256	6.9266	47.2776
	0.2	3.6632	13.3846	49.0312
	0.4	2.0809	24.4448	50.8695
3	0.1	7.2003	9.3161	67.0799
	0.2	4.0140	17.8873	71.8011
	0.4	2.9373	26.4353	77.6459

**Table 6:** Numerical Fisher information for $m=\alpha =l_{0}=0.1$.

*n*	ω	$I_{x}$	$I_{p}$	$I_{x}I_{p}$
1	0.1	6.4262	5.8528	37.6117
	0.5	14.6621	5.2390	76.8151
	1.0	21.1215	3.9753	83.9649
2	0.1	6.8256	6.9266	47.2776
	0.5	16.6462	4.6380	77.2062
	1.0	24.9990	3.7012	92.5259
3	0.1	7.2003	9.3161	67.0799
	0.5	18.3736	4.7996	2.87208
	1.0	28.2547	3.6296	102.5550

**Table 7:** Numerical Fisher–Shannon complexity information for $m=l_{0}=\omega =0.1$.

*n*	α	$C_{\mathrm{IS}}^{x}$	$C_{\mathrm{IS}}^{p}$
1	0.1	1.3214	128.8110
	0.3	1.3920	75.6725
	0.5	1.4215	57.8785
2	0.1	1.5345	201.6346
	0.3	1.6887	142.2901
	0.5	1.4846	220.4198
3	0.1	1.7284	201.3073
	0.3	1.9516	163.4668
	0.5	2.2868	147.9371

**Table 8:** Numerical Fisher–Shannon complexity information for m=α=ω=0.1.

*n*	$l_{0}$	$C_{\mathrm{IS}}^{x}$	$C_{\mathrm{IS}}^{p}$
1	0.1	1.3214	128.8110
	0.2	1.3253	171.1864
	0.4	1.3446	296.2610
2	0.1	1.5345	201.6346
	0.2	1.5681	204.4114
	0.4	1.8808	160.9670
3	0.1	1.7284	201.3073
	0.2	1.8015	318.4582
	0.4	2.1577	277.2870

**Table 9:** Numerical Fisher–Shannon complexity information for $m=\alpha =l_{0}=0.1$.

*n*	ω	$C_{\mathrm{IS}}^{x}$	$C_{\mathrm{IS}}^{p}$
1	0.1	1.3214	128.8110
	0.5	1.3263	152.0035
	1.0	1.3318	145.9913
2	0.1	1.5345	201.6346
	0.5	1.5764	140.7085
	1.0	1.6092	129.0467
3	0.1	1.7284	201.3073
	0.5	1.8186	115.3530
	1.0	1.8847	114.5429

## Data Availability

Data sharing is not applicable to this article as no new data were created or analyzed in this study.

## Appendix A. Detailed Derivation from Equation (3) to Equation (7)

In this Appendix we provide additional intermediate steps to clarify the derivation of Equation (7) from Equation (3), as requested by the referee. The main point concerns the explicit action of the fractal momentum operator and the cancellation of the additional terms through the appropriate choice of the effective potential.

We start from the stationary Schrödinger equation associated with the Hamiltonian operator in Equation (3), written schematically as

(A1) $${\hat{H}}^{\left(\alpha \right)}\psi \left(x\right)=E\psi \left(x\right),$$

where the fractal momentum operator is defined as

(A2) $${\hat{p}}^{\left(\alpha \right)}=-i\hbar {\left(\frac{l_{0}}{l-x}\right)}^{\alpha -1}\frac{d}{dx}.$$

Acting twice with the fractal momentum operator generates

(A3) $$\begin{array}{cc} {\hat{p}}^{(\alpha )2}\psi \left(x\right) & =-\hbar^{2}{\left(\frac{l_{0}}{l-x}\right)}^{2(\alpha -1)}\psi^{\prime \prime }\left(x\right)\\ \; & \;-\hbar^{2}\left(\alpha -1\right){\left(\frac{l_{0}}{l-x}\right)}^{2(\alpha -1)}\frac{1}{l-x}\psi^{\prime }\left(x\right), \end{array}$$

where the second term arises from the derivative acting on the position-dependent prefactor.

Including the position-dependent mass term in the kinetic energy operator, the Schrödinger equation takes the general form

(A4) $$-\frac{\hbar^{2}}{2m_{e}\left(x\right)}\left[{\left(\frac{l_{0}}{l-x}\right)}^{2(\alpha -1)}\psi^{\prime \prime }\left(x\right)+\frac{\alpha -1}{l-x}{\left(\frac{l_{0}}{l-x}\right)}^{2(\alpha -1)}\psi^{\prime }\left(x\right)\right]+V_{\mathrm{eff}}\left(x\right)\psi \left(x\right)=E\psi \left(x\right),$$

where $V_{\mathrm{eff}}\left(x\right)$ contains both the external potential and the von Roos contribution associated with the ordering ambiguity.

For the BenDaniel–Duke ordering, η=0, ξ=−1, ζ=0, the von Roos potential vanishes:

(A5) $$U_{\eta ,\xi ,\zeta }^{\mathrm{PDM}}\left(x\right)=0,$$

so that only the chosen external potential V(x) remains.

After dividing the entire equation by the prefactor $\left(\hbar^{2}/2m_{e}\left(x\right)\right){\left(l_{0}/\left(l-x\right)\right)}^{2(\alpha -1)}$, we obtain the equivalent differential equation

(A6) $$\psi^{\prime \prime }\left(x\right)+\frac{\alpha -1}{l-x}\psi^{\prime }\left(x\right)+\frac{2m_{e}\left(x\right)}{\hbar^{2}}{\left(\frac{l-x}{l_{0}}\right)}^{2(\alpha -1)}\left(E-V\left(x\right)\right)\psi \left(x\right)=0.$$

Finally, inserting the explicit form of the mass profile

(A7) $$m_{e}\left(x\right)=m{\left(\frac{l-x}{l_{0}}\right)}^{2(\alpha -1)},$$

and simplifying powers of $\left(l-x\right)/l_{0}$, we arrive at Equation (7):

(A8) $$\psi^{\prime \prime }\left(x\right)+\left[\frac{\alpha -1}{l-x}-{\left(\frac{l_{0}}{l-x}\right)}^{1-\alpha }\frac{m_{e}^{\prime }}{m_{e}}\right]\psi^{\prime }\left(x\right)+{\left(\frac{l_{0}}{l-x}\right)}^{2(1-\alpha )}\frac{2m_{e}}{\hbar^{2}}\left(E-V\right)\psi \left(x\right)=0.$$

This shows explicitly how the transformation arises from the action of the fractal kinetic operator and how the ordering choice eliminates the additional potential terms, leading to the final solvable form of the equation.

## References

[1] Moreira Q.R.D.S., Ximenes L.F., Moreira A.R.P., Silva J.B.R. (2026). Quantum information and thermodynamic features in position-dependent mass semiconductor heterostructures. Physica E.

[2] Bera A., Saha S., Ganguly J., Ghosh M. (2016). Noise-driven diamagnetic susceptibility of impurity doped quantum dots: Role of anisotropy, position-dependent effective mass and position-dependent dielectric screening function. Chem. Phys..

[3] Jana T.K. (2022). Supersymmetric Approach to Solve Interpolated Position—Dependent Mass Hamiltonians. Int. J. Sci. Res..

[4] von Roos O. (1983). Position-dependent effective masses in semiconductor theory. Phys. Rev. B.

[5] BenDaniel D.J., Duke C.B. (1966). Space-Charge Effects on Electron Tunneling. Phys. Rev. A.

[6] El-Nabulsi R.A. (2019). Some implications of position-dependent mass quantum fractional Hamiltonian in quantum mechanics. Eur. Phys. J. Plus.

[7] El-Nabulsi R.A. (2021). Position-dependent mass fractal Schrodinger equation from fractal anisotropy and product-like fractal measure and its implications in quantum dots and nanocrystals. Opt. Quant. Electron..

[8] Lima R.M., Christiansen H.R. (2023). Energy eigenstates of position-dependent mass particles in a spherical quantum dot. Eur. Phys. J. B.

[9] Christiansen H.R., Lima R.M. (2024). Effective particles in a multishell nanostructure with hardcore. Physica B.

[10] Christiansen H.R., Lima R.M. (2024). Three-dimensional bound states of cylindrical quantum heterostructures with position-dependent mass carriers. Phys. Scr..

[11] Valencia-Torres R., Avendaño J., García-Ravelo J., Choreño E. (2022). Position-dependent mass with modulated velocity in 1-D heterostructures. Phys. Scr..

[12] Peng Z.X., Li B.X., Deng C.S. (2025). Ultrahigh-Q Fano resonance in a cavity-waveguide coupled system based on second-order topological photonic crystals with elliptical holes. Opt. Laser Technol..

[13] Xu Y., Wang X., Wang Z. (2025). Quantum mapping algorithm for structural non-probabilistic reliability optimization. Struct. Multidiscip. Optim..

[14] Chen Y., Oberg L., Flick J., Lozovoi A., Meriles C.A., Doherty M.W. (2024). Semiempirical ab initio modeling of bound states of deep defects in semiconductor quantum technologies. Phys. Rev. B.

[15] Quesne C. (2023). Rational extensions of an oscillator-shaped quantum well potential in a position-dependent mass background. Phys. Scr..

[16] Mustafa O. (2023). Schrödinger oscillators in a deformed point-like global monopole spacetime and a Wu–Yang magnetic monopole: Position-dependent mass correspondence and isospectrality. Ann. Phys..

[17] Eyube E.S., Notani P.P., Wadata U., Najoji S.D., Bitrus B.M., Yabwa D., Tanko P.U. (2023). Energy spectrum and zero-temperature magnetic functions of a position-dependent mass system in a Pöschl-Teller-type potential constrained by a vector magnetic potential field. Phys. Scr..

[18] Jafarov E.I., Nagiyev S.M., der Jeugt J.V. (2026). Impact of the non-canonical approach to the exact solution of the ideal one-dimensional electron gas confined with an anisotropic quantum wire of oscillator-shaped profile. Eur. Phys. J. Plus.

[19] Jafarov E.I., Nagiyev S.M., der Jeugt J.V. (2025). Deformation of the Heisenberg–Weyl algebra and the Lie superalgebra osp(1|2): Exact solution for the quantum harmonic oscillator with a position-dependent mass. Eur. Phys. J. Plus.

[20] Karmand M., Amini M., Soltani M., Ghanbari-Adivi E., Jafari S.A. (2025). Solid-state analog of gravitational redshift: Transport signatures of massless Dirac fermions in tilted Dirac cone heterostructures. Phys. Rev. Res..

[21] Estrada-Delgado M.I., Blanco-Garcia Z.I. (2025). Oscillator Algebra in Complex Position-Dependent Mass Systems. Symmetry.

[22] Bagarello F., Balistreri E., Faddetta A. (2026). A position dependent mass Hamiltonian and abstract ladder operators. J. Phys. A.

[23] Timm L., Rüffert L.A., Weimer H., Santos L., Mehlstäubler T.E. (2021). Quantum nanofriction in trapped ion chains with a topological defect. Phys. Rev. Res..

[24] Batoul A.E., Moumene I., Oulne M., Adri M.E., Bassem Y.E. (2025). Phenomenological studies for *α*-decay process within position-dependent effective mass. Nucl. Phys. A.

[25] Sarathi P., Rawat B.S. (2025). Exact solution of Schrödinger equation for the complex Morse potential to investigate physical systems with position-dependent complex mass. Phys. Scr..

[26] Ruby V.C., Lakshmanan M. (2025). A quantum approach to the continuum Heisenberg spin-chain model: Position-dependent mass formalism and pre-canonical quantization. J. Math. Phys..

[27] Mendez M., Pont F.M. (2025). Dynamics of correlations and entanglement generation in electron-molecule inelastic scattering. Phys. Rev. A.

[28] Lawson L.M., Amouzouvi K., Sodoga K., Beltako K. (2024). Position-dependent mass from noncommutativity and its statistical descriptions. Int. J. Geom. Meth. Mod. Phys..

[29] Liu J., Ma C., Li M., He J., Hua C., Wang L., Yang J., Liu K., Zhou Y. (2026). Physics-informed hybrid digital twin framework integrating fractal contact modeling and edge-cloud artificial intelligence for dynamic thermal contact conductance prediction. Int. J. Heat Mass Transf..

[30] Li Z., Shu Y., Lu L., Sun J., Shen Q., Gao P., Zdańkows P., Trusiak K., Kujawinska M., Chen Q. (2025). Fourier Holo-Ptychographic Microscopy: A Hybrid Digital Holography-Fourier Ptychography Approach to Synthetic-Aperture Quantitative Phase Microscopy. ACS Photonics.

[31] Billing G.D. (2003). The Quantum Classical Theory: More Complex Systems.

[32] Konishi E. (2020). Holographic interpretation of Shannon entropy of coherence of quantum pure states. Europhys. Lett..

[33] Li T., Li M., Huang J. (2016). Quantum Fisher information of triphoton states. Chin. Opt. Lett..

[34] Arbekov I.M., Molotkov S.N. (2017). Distinguishability of quantum states and shannon complexity in quantum cryptography. J. Exp. Theor. Phys..

[35] Serrano F.A., Falaye B.J., Dong S.H. (2016). Information-theoretic measures for a solitonic profile mass Schrödinger equation with a squared hyperbolic cosecant potential. Physica A.

[36] Sun G.H., Dong S.H. (2013). Quantum information entropies of the eigenstates for a symmetrically trigonometric Rosen–Morse potential. Phys. Scr..

[37] Shi Y.J., Sun G.H., Jing J., Dong S.H. (2017). Shannon and Fisher entropy measures for a parity-restricted harmonic oscillator. Laser Phys..

[38] Lima F.C.E., Moreira A.R.P., Almeida C.A.S. (2021). Information and thermodynamic properties of a non-Hermitian particle ensemble. Int. J. Quant. Chem..

[39] Lima F.C.E., Moreira A.R.P., Almeida C.A.S. (2021). Statistical properties of linear Majorana fermions. Int. J. Quant. Chem..

[40] Ibl M., Čapek J. (2016). Measure of Uncertainty in Process Models Using Stochastic Petri Nets and Shannon Entropy. Entropy.

[41] American Chemical Society (ACS) (2024). Supporting Information for Fisher Oscillations.

[42] Stephens S. (2017). Heisenberg: The Uncertainty Principle.

[43] Ikhdair S.M. (2012). Effective Schroedinger equation with general ordering ambiguity position-dependent mass Morse potential. Mol. Phys..

[44] Roy-Layinde T.O., Vincent U.E., Abolade S.A., Popoola O.O., Laoye J.A., McClintock P.V.E. (2021). Vibrational resonances in driven oscillators with position-dependent mass. Philos. Trans. R. Soc. A.

[45] Benter S., Dubrovskii V.G., Bartmann M., Campo A., Zardo I., Sistani M., Stöger-Pollach M., Lancaster S., Detz H., Lugstein A. (2019). Quasi One-Dimensional Metal–Semiconductor Heterostructures. Nano Lett..

[46] Elizondo S.L., Shi Z. (2007). One-dimensional analysis of *N*-on-*p* Pb_1−x_Sn_x_Se compositionally graded heterojunction photodetectors. J. Appl. Phys..

[47] Tian H., Ji Y., Li C., Liu H. (2007). Transmission properties of one-dimensional graded photonic crystals and enlargement of omnidirectional negligible transmission gap. Opt. Commun..

[48] Xiong X., Zheng J., Huang J., Yang Y., Huang X., Liu C. (2026). Recent Advances in Two-Dimensional Metallic MXenes as High-Performance Saturable Absorbers. Nanomaterials.

[49] Batle J., Ciftja O., Aty M.A., Hafez M.A., Alkhazaleh S. (2024). Effective Piecewise Mass Distributions for Optimal Energy Eigenvalues of a Particle in Low-Dimensional Heterojunctions. Nanomaterials.

[50] Donado F.A., Almanza F.G., Viña C.F., Vinasco J.A., Sierra-Ortega J., Escorcia-Salas G.E., Hahn R.V.H., Mora-Ramos M.E., Mommadi O., Moussaouy A.E. (2025). Impact of Nonresonant Intense Laser and Electric Fields on a Low-Dimensional CdTe/CdSe Type-II Cone. Nanomaterials.

[51] Shannon C.E. (1948). A Mathematical Theory of Communication. Bell Syst. Tech. J..

[52] Beckner W. (1975). Inequalities in Fourier Analysis. Ann. Math..

[53] Bialynicki-Birula I., Mycielski J. (1975). Uncertainty relations for information entropy in wave mechanics. Commun. Math. Phys..

[54] Fisher R.A. (1925). Theory of Statistical Estimation. Math. Proc. Camb. Philos. Soc..

[55] Romera E., Sanchez-Moreno P., Dehesa J.S. (2005). The fisher information of single-particle systems with a central potential. Chem. Phys. Lett..

[56] Dembo A., Cover T.M., Thomas J.A. (1991). Information theoretic inequalities. IEEE Trans. Inf. Theory.

